# CCN2 Is Required for the TGF-β Induced Activation of Smad1 - Erk1/2 Signaling Network

**DOI:** 10.1371/journal.pone.0021911

**Published:** 2011-07-08

**Authors:** Sashidhar S. Nakerakanti, Andreea M. Bujor, Maria Trojanowska

**Affiliations:** The Arthritis Center, Boston University School of Medicine, Boston, Massachusetts, United States of America; University of Birmingham, United Kingdom

## Abstract

Connective tissue growth factor (CCN2) is a multifunctional matricellular protein, which is frequently overexpressed during organ fibrosis. CCN2 is a mediator of the pro-fibrotic effects of TGF-β in cultured cells, but the specific function of CCN2 in the fibrotic process has not been elucidated. In this study we characterized the CCN2-dependent signaling pathways that are required for the TGF-β induced fibrogenic response. By depleting endogenous CCN2 we show that CCN2 is indispensable for the TGF-β-induced phosphorylation of Smad1 and Erk1/2, but it is unnecessary for the activation of Smad3. TGF-β stimulation triggered formation of the CCN2/β_3_ integrin protein complexes and activation of Src signaling. Furthermore, we demonstrated that signaling through the α_v_β_3_ integrin receptor and Src was required for the TGF-β induced Smad1 phosphorylation. Recombinant CCN2 activated Src and Erk1/2 signaling, and induced phosphorylation of Fli1, but was unable to stimulate Smad1 or Smad3 phosphorylation. Additional experiments were performed to investigate the role of CCN2 in collagen production. Consistent with the previous studies, blockade of CCN2 abrogated TGF-β-induced collagen mRNA and protein levels. Recombinant CCN2 potently stimulated collagen mRNA levels and upregulated activity of the COL1A2 promoter, however CCN2 was a weak inducer of collagen protein levels. CCN2 stimulation of collagen was dose-dependent with the lower doses (<50 ng/ml) having a stimulatory effect and higher doses having an inhibitory effect on collagen gene expression. In conclusion, our study defines a novel CCN2/α_v_β_3_ integrin/Src/Smad1 axis that contributes to the pro-fibrotic TGF-β signaling and suggests that blockade of this pathway may be beneficial for the treatment of fibrosis.

## Introduction

TGF-β is a multifunctional polypeptide growth factor that regulates cell proliferation, functional differentiation, extracellular matrix (ECM) production, cell motility, and apoptosis [1]. Canonical TGF-β signaling is initiated by ligand binding to a heteromeric complex of transmembrane serine/threonine kinases, type I (ALK5) and type II, and subsequent activation of transcriptional co-regulators, Smad2 and Smad3 [1]. In addition, several recent studies have shown that TGF-β can also activate Smad1/5 signaling [2], [3], [4]. In endothelial cells, this mode of signaling involves ALK5 and ALK1 receptors, and also depends on an accessory receptor, endoglin [3], [5]. However in other cell types including various epithelial cell lines, Smad1/5 is phosphorylated by ALK5 receptor independently of BMP receptors [4], [6]. Besides activation of Smad pathways, TGF-β induces numerous other signaling molecules, including MAP kinases, PI3 kinase/Akt, and Rho-like GTPase [7], [8]. Deregulated TGF-β signaling has been implicated in various pathological conditions, including fibrosis and cancer.

Connective Tissue Growth Factor (CTGF, CCN2) is a member of the CCN family of matricellular proteins, which play important roles in a variety of cellular processes, including angiogenesis, chondrogenesis, and wound healing [9]. CCN2 expression is also frequently deregulated during pathological conditions such as fibrosis and cancer [10], [11]. In particular, overexpression of CCN2 has been demonstrated in a number of fibrotic diseases occurring in different organs, strongly suggesting an important role for this growth factor in the process of excessive matrix deposition [12]. Transgenic mice overexpressing CCN2 in fibroblasts developed fibrosis in multiple organs [13], whereas mice lacking fibroblast expression of CCN2 were protected from the bleomycin-induced dermal fibrosis [14]. Recent genetic evidence further supports a role for CCN2 in fibrosis [15], [16]. Consistent with this view, it has been shown that CCN2 synthesis is induced by TGF-β and that it is required for the TGF-β induction of collagen [17]. Specific mechanisms involved in the CCN2-dependent fibrogenic response have not been elucidated. In general, the intracellular signaling elicited by the members of the CCN family, including CCN2, remains elusive, because the *bona fide* CCN receptor has not been identified. However, it has been well documented that CCN2 interacts with various integrin receptors in a cell-type dependent manner. For example, adhesion of CCN2 to the α_6_β_1_ integrin receptor and heparan sulphate proteoglycan leads to activation of ERK1/2 and upregulation of MMP1 in fibroblasts, [18], while in endothelial cells CCN2 promotes angiogenic responses through binding to the α_v_β_3_ integrin [19]. Similarly, α_v_β_3_ integrin is required for the CCN2 induced migration of mesangial cells [20]. Furthermore, activation of Erk1/2, PKB, and Src and upregulation of fibronectin by CCN2 is also dependent on β_3_ integrin in mesangial cells [20]. Other signaling molecules, which were shown to be activated in mesangial cells by CCN2 include JNK, CaMKII, PKCα and PKCδ [21]. Consistent with these findings, it has been reported that CCN2 signals through neurotrophin receptor TrkA, suggesting an ability to cross-activate receptors with a tyrosine kinase activity (RTK) [21], but so far this observation has not been extended to other RTKs. It has also been suggested that CCN2 exerts its biological effects through modulating the activity of other growth factors. For example, Abreu *et al* have shown that CCN2 binds to TGF-β resulting in increased ligand receptor binding, whereas CCN2 binding to BMP4 interferes with the BMP4 receptor binding [22]. Despite the progress in identifying signaling pathways elicited by CCN2, the specific requirement for CCN2 in the TGF-β-dependent up-regulation of collagen genes remains unexplained.

Because CCN2 is considered a key mediator of the fibrogenic effects of TGF-β and is viewed as a potential target for the anti-fibrotic therapies, we wished to gain additional insights into the molecular mechanisms governing the fibrogenic activity of CCN2. Here we show that CCN2 is required for the TGF-β induced phosphorylation of Smad1, Erk1/2 and Fli1, while it is dispensable for the activation of Smad3 signaling. These effects of CCN2 are mediated through the α_v_β_3_ integrin receptor and also involve activation of nonreceptor tyrosine kinase Src. However, in the absence of TGF-β, CCN2 is not able to activate Smad1 signaling and is only a weak inducer of collagen protein in dermal fibroblasts.

## Results

### CCN2 mediates TGF-β induced collagen expression

**Figure 1:**
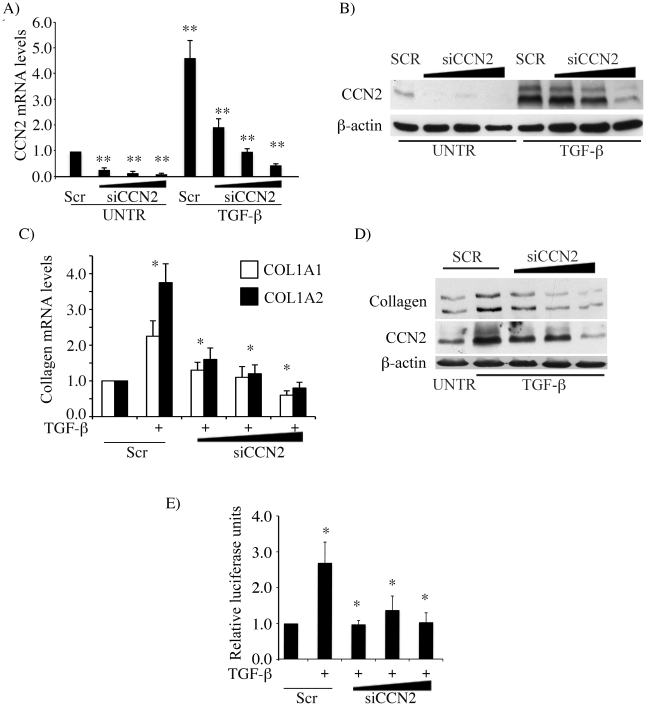
CCN2 mediates TGF-β induced collagen upregulation. (A, B) Foreskin fibroblasts were transduced with increasing doses of AdenoCCN2 siRNA virus or the control scramble virus for 72 hours. CCN2 mRNA levels were analyzed by quantitative RT-PCR (A) and the protein levels by western blot (B). (C,D) Foreskin fibroblasts were transduced with increasing doses of AdenoCCN2 siRNA virus or the control scramble virus for 72 hours followed by stimulation with TGF-β for 48 hours. Collagen mRNA levels were analyzed by quantitative RT-PCR (C) and collagen protein levels by western blot (D). (E) Foreskin fibroblasts were transduced with increasing doses of AdenoCCN2 siRNA virus or the control scramble virus for 72 hours followed by transfection with COL1A2 (−2 Kb) luciferase promoter plasmid construct. Next day after transfection TGF-β was added for 24 hours and the promoter activity was determined. The values represent mean ± S.E. of three independent experiments. * & **significant values at *p*<0.05 & *p*<0.001 respectively.

In the first set of experiments, we established an experimental model to investigate the role of CCN2 in TGF-β signaling. In agreement with previous studies using different cell types [23], [24], [25], [26], in foreskin fibroblasts TGF-β induction of CCN2 mRNA and protein preceded that of collagen, whereas up-regulation of collagen persisted for a longer time (data not shown). To study the role of CCN2 in the TGF-β induced up-regulation of collagen, endogenous CCN2 was knocked down using adenoviral CCN2 siRNA. Transduction with CCN2 siRNA virus efficiently suppressed the endogenous levels of CCN2 by more than 80% both at the mRNA and protein level (Fig. 1A-B). Consistent with previous reports, depletion of CCN2 almost completely abrogated TGF-β induced up-regulation of collagen synthesis (Fig. 1C-D). Furthermore, transcriptional activation of COL1A2 promoter by TGF-β was inhibited in the absence of CCN2 (Fig. 1E). These results are in agreement with the previously published reports that implicated CCN2 in the TGF-β induced collagen gene expression [17], [27], [28], [29].

### CCN2 is required for the TGF-β induced activation of the Smad1 pathway

**Figure 2:**
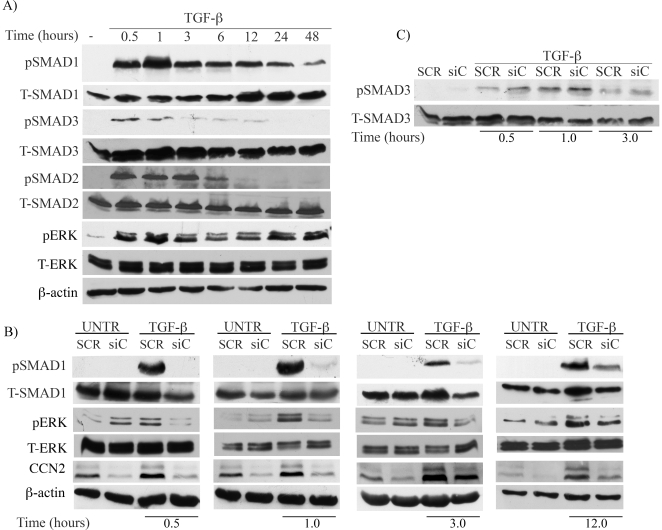
CCN2 is required for the TGF-β induced activation of Smad1 and Erk1/2 pathways. (A) Foreskin fibroblasts were stimulated with TGF-β for the indicated time periods and analyzed for Smad1, Smad3, Smad2 and Erk phosphorylation kinetics. (B,C) Fibroblasts were transduced with AdenoCCN2 siRNA or the control scrambled virus for 72 h followed by stimulation with TGF-β for the indicated time points. Phosphorylation status and the total protein levels of Smad1, Erk1/2, and Smad3 were determined by western blot.

We next sought to determine the role of CCN2 in mediating TGF-β induction of collagen. Previous studies have shown that in addition to the classical Smad3 pathway, the non-Smad3 pathways such as MAP Kinase, as well as Smad1 play a role in the TGF-β regulation of collagen gene expression [3]. We first determined the kinetics of the activation of Smad1, Smad2, Smad3, and Erk1/2 pathways in response to TGF-β stimulation. TGF-β induced rapid phosphorylation of Smad1, Smad2, Smad3 and Erk1/2 proteins within 30 minutes post stimulation (Fig. 2A). Activation of Smad1 and Erk1/2 pathways was sustained up to 48 hours, while activation of Smad2 and Smad3 was more transient and was not detectable after 24 hours. We have also observed an increase in Smad1 protein levels starting at 12 hours post stimulation. We next investigated whether CCN2 contributes to activation of the above signaling molecules. CCN2 was depleted from the cells using adenoviral siRNA prior to stimulation with TGF-β. As shown in Fig. 2B, phosphorylation of Smad1 in response to TGF-β was almost completely abolished in the absence of CCN2. Up-regulation of total Smad1 protein levels was also abrogated. Likewise, TGF-β induced phosphorylation of Erk1/2 was significantly decreased (Fig. 2B and Fig. S1A). On the other hand, depletion of CCN2 had no appreciable effect on the TGF-β induced Smad3 phosphorylation (Fig. 2C and Fig. S1B), in agreement with the recently reported observations by Quan et al (2011) [30]. We concluded from these experiments that CCN2 is required for the TGF-β-induced Smad1 and Erk1/2 phosphorylation.

### CCN2/α_v_β_3_ integrin signaling contributes to the TGF-β dependent activation of the Smad1 pathway

**Figure 3:**
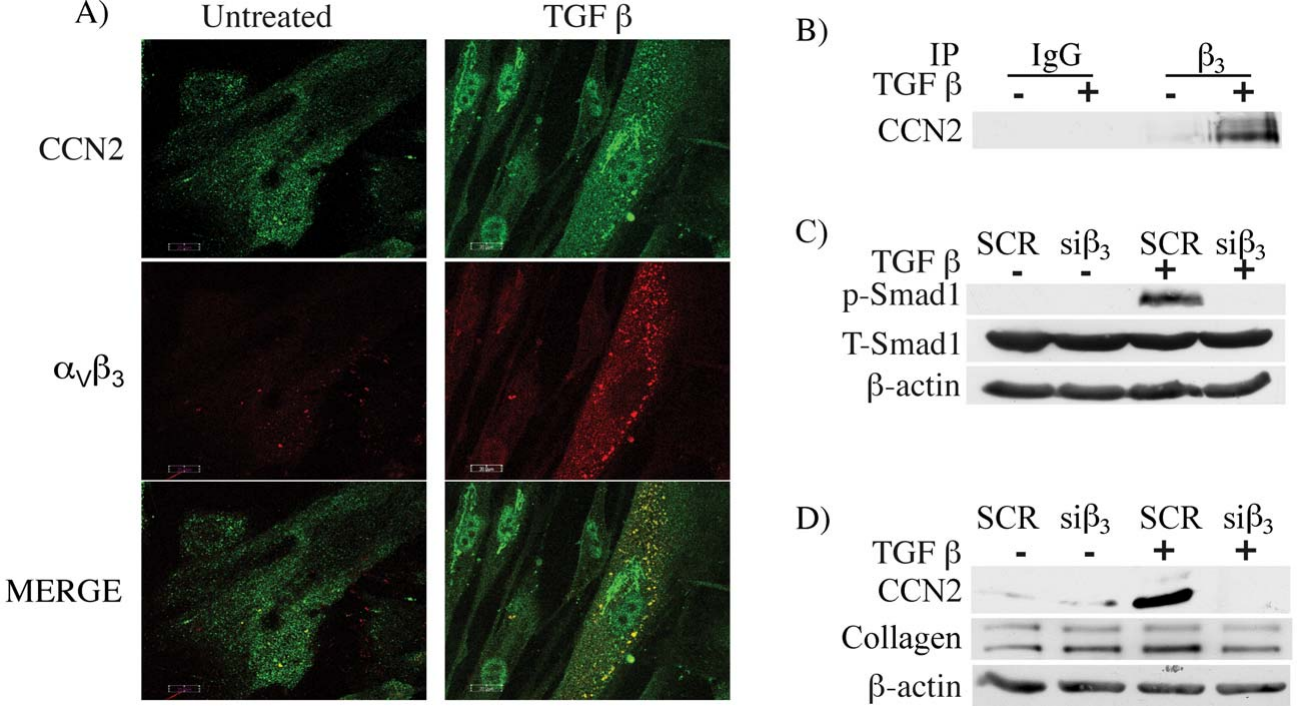
TGF-β induced Smad1 phosphorylation is mediated through α_v_β_3_ integrin. (A) Foreskin fibroblasts cultured on cover slips were stimulated with TGF-β for 30 minutes. The cells were fixed with paraformaldehyde and incubated with CCN2 (green) and α_v_β_3_ (red) antibodies and analyzed by Confocal microscopy. (B) Cell lysates from TGF-β stimulated cells were immunoprecipitated with β_3_ integrin and analyzed for CCN2 by western blot. (C) Fibroblasts were transfected with β_3_ siRNA oligos and then stimulated with TGF-β for 30 minutes and analyzed for Smad1 phosphorylation by western blot. (D) Fibroblasts were transfected with β_3_ siRNA oligos and then stimulated with TGF-β for 24 hours and examined for CCN2 and Collagen levels.

Previous reports have demonstrated that CCN2 signals via integrin receptors and heparan sulfate proteoglycan (HSPG). We focused on the α_v_β_3_ integrin, which was shown to cluster with the TGF-βRII and to mediate TGF-β induced proliferation in lung fibroblasts [31]. Importantly, α_v_β_3_ integrin was also shown to mediate the profibrotic effects of TGF-β in scleroderma fibroblasts [32]. We used immunofluorescence to examine the distribution of CCN2 and α_v_β_3_ integrin in response to TGF-β. In unstimulated cells, CCN2 and α_v_β_3_ integrin were diffusely distributed in the cytoplasm (Fig. 3A), while after 30 minutes of treatment with TGF-β, co-localization of CCN2 with α_v_β_3_ integrin was observed. To further verify these findings we used co-immunoprecipitation. As shown in Fig. 3B formation of CCN2 complexes with β_3_ integrin was observed in cells stimulated for 30 min. with TGF-β, but was not detectable in unstimulated fibroblasts. To determine whether α_v_β_3_ integrin contributes to Smad1 activation in response to TGF-β, we suppressed the endogenous levels of β_3_ integrin >70% using siRNA (Fig. S2A). As shown in Fig. 3C, depletion of β_3_ integrin abrogated TGF-β induced phosphorylation of Smad1, suggesting that α_v_β_3_ integrin receptor signaling contributes to the TGF-β mediated activation of Smad1 pathway. We also observed similar decrease in TGF-β induced phosphorylation of Smad1 when we blocked α_v_β_3_ integrin function using LM609 antibody prior to fibroblast stimulation with TGF-β (Fig. S2B). Furthermore, depletion of β_3_ integrin levels abrogated TGF-β induced expression of CCN2 and moderately decreased expression of collagen (Fig. 3D). To investigate whether HSPG is also involved in activation of Smad1, fibroblasts were treated with Xylosidase to block HSPG function, prior to stimulation with TGF-β. This treatment did not affect phosphorylation of Smad1, suggesting that HSPG is not involved in this process (data not shown).

### Src is required for the TGF-β dependent activation of Smad1 pathway

**Figure 4:**
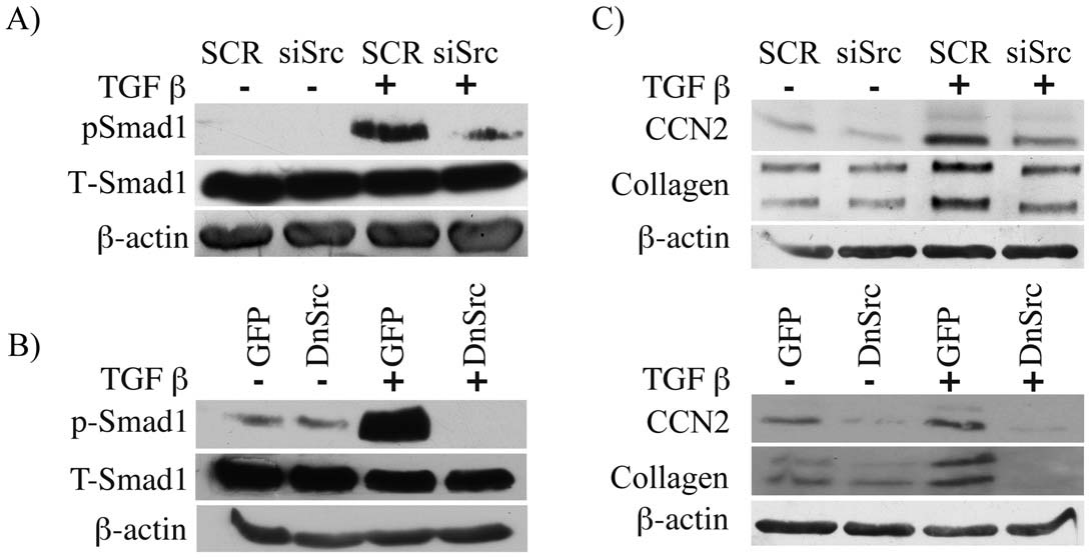
Src mediates TGF-β induced Smad1 phosphorylation. (A) Foreskin fibroblasts were transfected with control (SCR) or Src siRNA oligos. pSmad1 and total Smad1 were analyzed by western blot. (B) Foreskin fibroblasts were transduced with dominant negative Src adenovirus and then stimulated with TGF-β for 30 minutes. pSmad1 and total Smad1 were examined by western blot. (C) Foreskin fibroblasts were transfected with Src siRNA oligos (top panel) or transduced with dominant negative Src adenovirus (lower panel), then stimulated with TGF-β for 24 hours and examined for CCN2 and collagen levels by western blot.

Src is one of the major nonreceptor tyrosine kinases activated downstream of both α_v_β_3_ integrin and TGF-β [33], [34]. In addition, studies by Crean et al (2004) showed that Src is rapidly induced by CCN2 [20]. To determine if Src is involved in activation of Smad1 signaling, we blocked Src activity using three independent approaches: knock down by Src siRNA (Fig. S3A), forced expression of a dominant negative Src, and a specific pharmacological inhibitor of Src, SU6656 (Fig S3B). Blockade of Src abrogated TGF-β stimulation of Smad1 phosphorylation (Fig. 4A-B). Furthermore, blockade of Src attenuated TGF-β induced up-regulation of CCN2 and collagen (Fig. 4C). Taken together, these results suggest that TGF-β induced activation of Smad1 and its target genes requires CCN2, α_v_β_3_ integrin, and Src.

### CCN2 activates Src and Erk1/2 pathways in dermal fibroblasts

**Figure 5:**
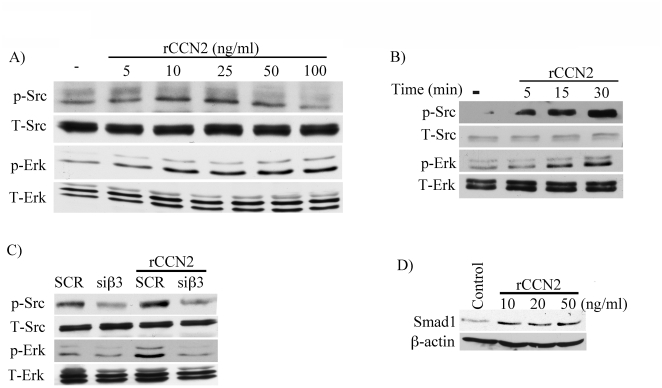
CCN2 induces Src and Erk1/2 phosphorylation. (A) Foreskin fibroblasts were serum starved overnight and then stimulated with indicated doses of recombinant CCN2 (rCCN2) (5 - 100 ng/ml) for 30 minutes and analyzed for Src and Erk1/2 phosphorylation. (B) Foreskin fibroblasts were serum starved overnight and then stimulated with rCCN2 (25 ng/ml) for the indicated time points and analyzed for Src and Erk1/2 phosphorylation. (C) Foreskin fibroblasts were transfected with β3 siRNA oligos and then stimulated with rCCN2 for 30 minutes and examined the Src and Erk1/2 phosphorylation by western blot. (D) Foreskin fibroblasts were treated with rCCN2 for 48 hours and then were analyzed for Smad1 expression levels by western blot.

Thus far we established that CCN2 is required for the activation of selected TGF-β induced signaling pathways, including Smad1 and Erk1/2; we next sought to determine whether CCN2 alone is sufficient to induce these pathways. Treatment of cells with recombinant human CCN2 (rCCN2) (5–100 ng/ml) for 30 min. induced phospho-Src in a dose-dependent manner with maximal stimulation observed at 25 ng/ml and no stimulatory effects at a higher dose (100 ng/ml), while phospho-Erk1/2 showed a different pattern with activation at all the doses tested (5–100 ng/ml. (Fig. 5A). In a time course experiment, rCCN2 (25 ng/ml) rapidly induced phosphorylation of Src (5 min.) with maximal induction observed at 30 min, while Erk1/2 activation occurred with delayed kinetics as compared to Src (Fig. 5B). To determine whether β3 integrin is required for the CCN2 stimulation of p-Src and p-Erk1/2, β3 integrin was knockdown using siRNA. As shown in Fig 5C, depletion of β3 decreased basal and CCN2 induced phosphorylation of Src and Erk1/2. In contrast to Src and Erk1/2, treatment with CCN2 did not stimulate Smad1 or Smad3 phosphorylation (data not shown), albeit CCN2 moderately increased total Smad1 levels (Fig. 5D and Fig. S4). Together, these data suggest that CCN2 via β3 integrin is sufficient to activate selected TGF-β inducible pathways, including Src and Erk1/2, however CCN2 alone is not able to activate the Smad1 pathway.

### CCN2 is a moderate inducer of collagen synthesis

**Figure 6:**
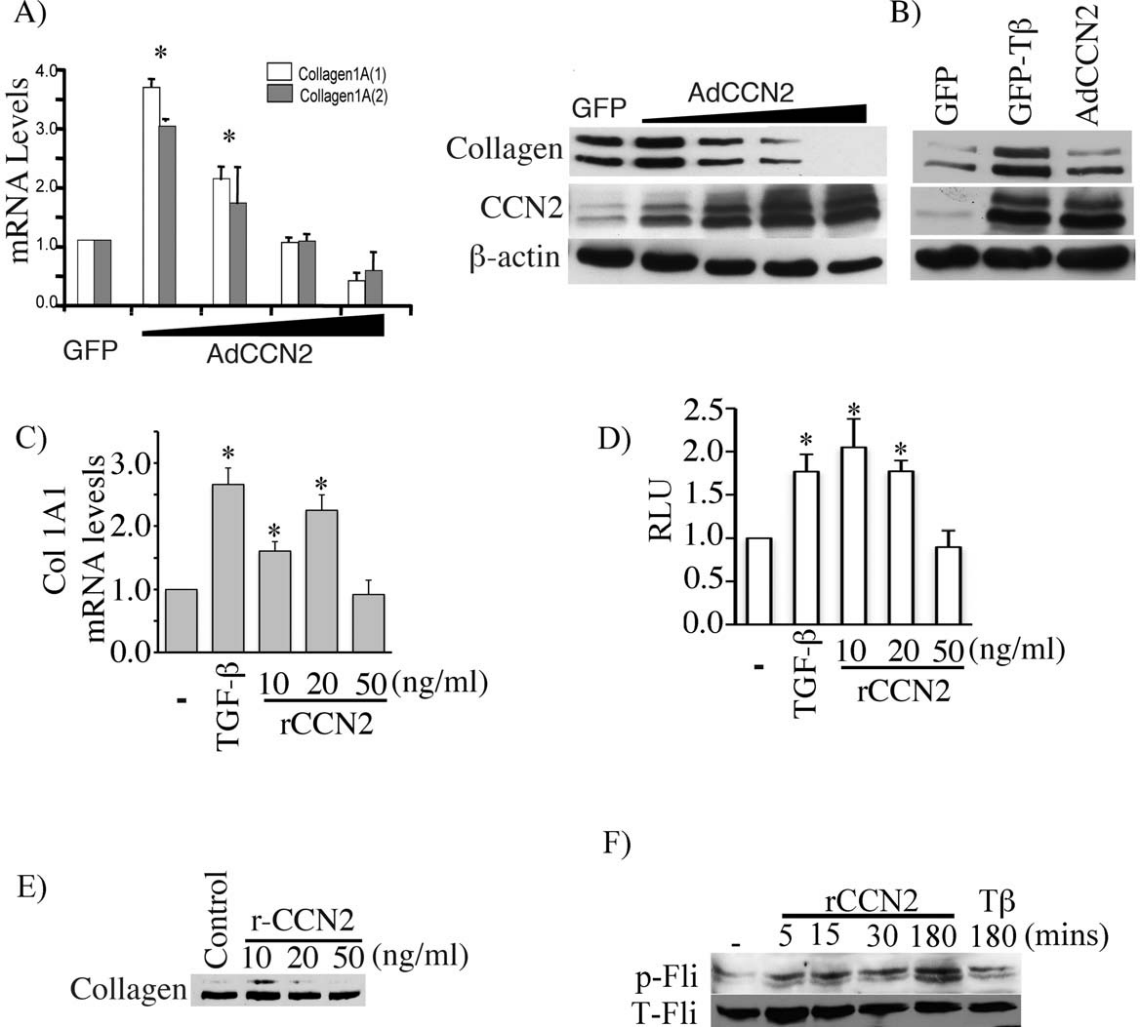
CCN2 induced up-regulation of collagen gene expression is dose dependent. (A) Foreskin fibroblasts were transduced with increasing doses of CCN2 (5, 10, 25, 50 MOI) overexpressing virus. Collagen mRNA levels were determined by quantitative RT-PCR (A, left panel) and collagen protein levels were determined by western blot (A, right panel). The values represent mean ± S.E. of three independent experiments. *, significant values at *p*<0.05. (B) Foreskin fibroblasts were stimulated with 2.5 ng/ml of TGF-β or transduced with AdCCN2 (10 MOI) for 48 hours. Collagen and CCN2 protein levels were determined by western blot. (C) COL1A1 mRNA levels was determined by qPCR in foreskin fibroblasts stimulated with TGF-β (2.5 ng/ml) or indicated doses of rhCCN2 for 24 hours. The values represent mean ± S.E. (n = 4; *, significant values at p<0.05). (D) Foreskin fibroblasts were transfected with COL1A2 (−2 Kb) luciferase promoter plasmid construct. Next day after transfection TGF-β or rCCN2 were added for 24 hours and the promoter activity was determined. The values represent mean ± S.E. (n = 4; *, significant values at at p<0.05). (E) Foreskin fibroblasts were treated with rCCN2 at various concentrations and analyzed for collagen levels in the culture medium. (F) Foreskin fibroblasts were treated with rCCN2 (20 ng/ml) at the indicated time periods and with TGF-β (2.5 ng/ml) for 3 hours and analyzed for phosphorylated and total Fli1 by western blot from nuclear extracts.

To determine whether CCN2 alone is capable to induce collagen synthesis, we first used CCN2 expressing adenovirus. Foreskin fibroblasts were infected with increasing doses of virus. We observed that CCN2 modulates collagen synthesis in a dose-dependent fashion. Overexpression of CCN2 at moderate levels resulted in induction of collagen mRNA and protein, while high levels of CCN2 markedly decreased collagen mRNA and protein synthesis (Fig. 6A). We next compared side-by-side the effects of TGF-β and CCN2 on collagen production. By carefully titrating the dose of adenovirus, CCN2 was expressed at the level observed in control cells after TGF-β stimulation (Fig. 6B). Whereas both of these treatments increased collagen protein production, the response was more robust in cells stimulated with TGF-β vs cells overexpressing CCN2, suggesting that CCN2 alone is only a weak inducer of collagen protein as compared to TGF-β. Additional experiments were performed using rCCN2. Upregulation of COL1A1 mRNA level (Fig. 6C) and COL1A2 promoter activity (Fig. 6D) was comparable between rCCN2 and TGF-β, while collagen protein was only weakly induced by rCCN2 (Fig. 6E). These data suggest that CCN2 may be primarily involved in collagen regulation at the transcriptional level. Since recent studies have indicated that TGF-β stimulation of collagen gene expression requires inactivation of the transcriptional repressor Fli1 through a PKCδ-dependent Fli1 phosphorylation [35], we examined the effects of rCCN2 on Fli1 phosphorylation status. As a positive control, cells were stimulated for 3 hours with TGF-β as previously described [35]. Recombinant CCN2 rapidly and potently induced Fli1 phosphorylation to a much higher level than that observed after TGF-β stimulation (Fig. 6F). While these results suggest that CCN2 may function as a primary inducer of p-Fli1 in the TGF-β pathway, additional studies are required to further investigate this interesting finding. Collectively, these results indicate that effects of CCN2 are dose-dependent, with the pro-fibrotic effects observed only with the low doses (in the range of those induced by TGF-β).

## Discussion

**Figure 7:**
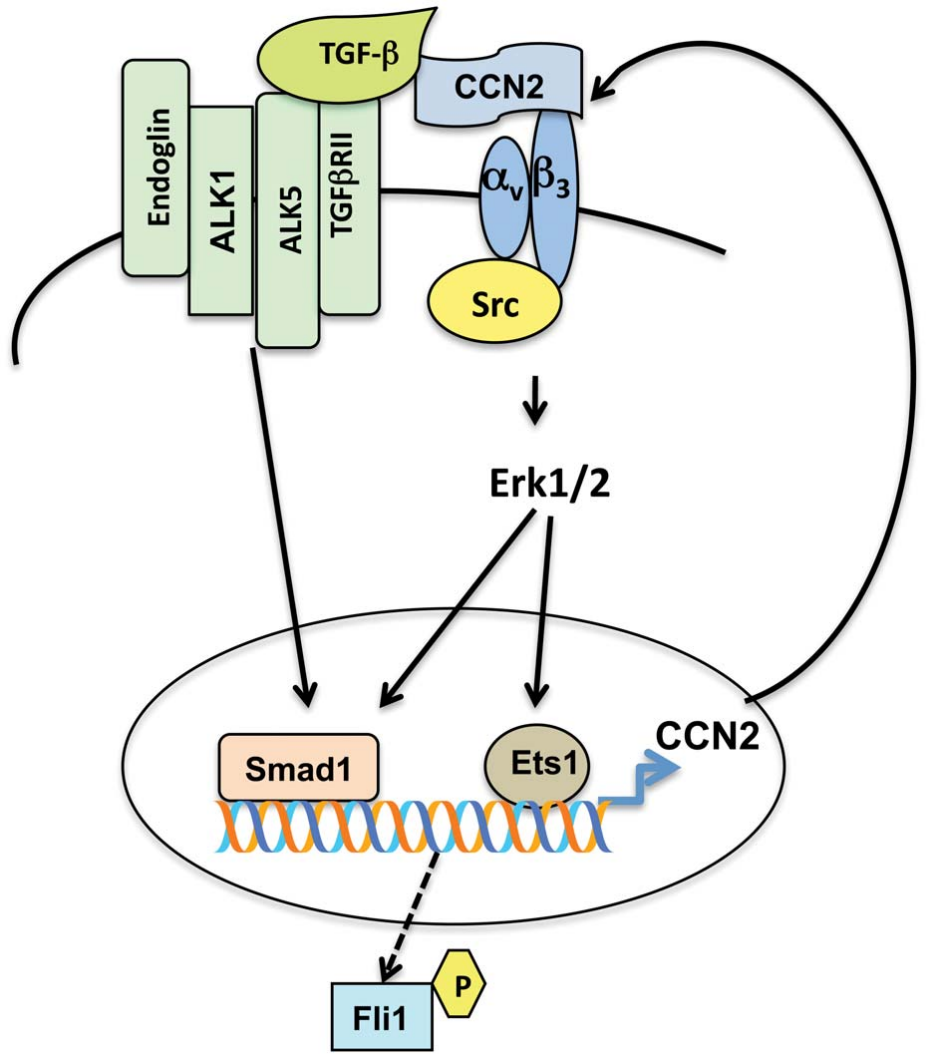
A proposed model for the positive feedback loop between TGF-β/Smad1 and CCN2 pathway. See Discussion text for details.

CCN2 is almost universally overexpressed during organ fibrosis and is considered to be a key effector of the fibrogenic effects of TGF-β. While the precise contribution of CCN2 to this process has not been clearly defined, previous studies support the view of cooperation between CCN2 and TGF-β in the process of sustained fibrotic response [22], [36]. In this study we focused on characterizing the CCN2-dependent signaling pathways that are required for the TGF-β induced fibrogenic process. By depleting endogenous CCN2 we show that CCN2 is necessary for the TGF-β induced phosphorylation of Smad1 and Erk1/2, but it is dispensable for activation of the Smad3 pathway. Our study suggests that this action of CCN2 is mediated via integrin receptor signaling. In response to TGF-β stimulation, CCN2 forms complexes with the α_v_β_3_ integrin receptor, which triggers activation of Src and Erk1/2 pathways, but the specific mechanisms involved in activation of these two pathways is presently unknown. In agreement with previous studies in mesangial cells [20], we show that CCN2 alone activates Src and Erk1/2 signaling. However, CCN2 is not able to phosphorylate either Smad1 or Smad3. Consequently, in the absence of TGF-β, CCN2 is only a weak inducer of collagen protein synthesis. Together, these data suggest that activation of Smad1 pathway requires coordinated action of TGF-β and CCN2 signaling through their cognate receptors. As CCN2 has been shown to bind TGF-β and enhance TGF-β receptor binding and signaling [22], Smad1 and Erk1/2 pathways could be activated in a spatially and temporally coordinated manner (Fig. 7).

This study revealed a dose-dependent effect of CCN2 on collagen gene regulation. The stimulatory effects were only observed at low doses of CCN2, in the range of those induced by TGF-β, while at high doses CCN2 inhibited basal collagen mRNA and protein expression. Furthermore, while we observed that CCN2 is quite potent in inducing collagen mRNA levels and collagen promoter activity, collagen protein levels were only weakly induced. Interestingly, we observed that rCCN2 induces rapid (5 min.) and sustained (up to 3 hours) phosphorylation of Fli1, which constitutes a key event regulating dissociation of Fli1 from the collagen promoter [35]. CCN2-mediated dissociation of Fli1 may be a primary mechanism of the transcriptional upregulation of collagen genes by CCN2, however other transcription factors, which are activated by Erk1/2 signaling e.g. Sp1 [37] and Erg-1 [38], could also be involved. Interestingly, however, an increase in collagen mRNA was not sufficient to result in significant changes in collagen protein production, suggesting that other cellular pathways, which are induced by TGF-β or other co-stimulators such as insulin/IGF-1 [39] are required for the significant upregulation of collagen protein by CCN2. In contrast to its contribution to the pro-fibrotic effects of TGF-β, CCN2 is a potent inducer of the collagen-degrading enzyme MMP1 [18]. Furthermore, our recent studies demonstrate that rCCN2 upregulates MMP1 gene expression through activation of key transcriptional regulators of this gene, including Erk1/2/Ets1 and c-Jun [40]. CCN2-induced MMP1 synthesis was not dose-sensitive and occurred at a wide range of CCN2 concentrations (50–500 ng/ml). On the other hand, profibrotic effects of CCN2 were observed only at the lower doses of CCN2 (<50 ng/ml). This observation adds further complexity to the molecular mechanisms underlying diverse roles of CCN2 in matrix regulation. Together, these data suggest that the role of CCN2 in matrix regulation is context-dependent and its profibrotic function is fully manifested only in the presence of TGF-β or other profibrotic mediators.

There is increasing evidence that activation of Smad1 signaling may play a pivotal role in the process of fibrosis [41], [42], [43]. Consistent with this notion, blockade of Smad1 via siRNA completely abrogated TGF-β stimulation of collagen and CCN2 in dermal fibroblasts [3]. The ability of TGF-β to activate Smad1 signaling, in addition to the conventional Smad2/3, has been confirmed in a variety of cells; however, specific mechanisms involved in this activation may differ in different cell types [4], [6]. TGF-β dependent activation of Smad1 in dermal fibroblasts has not been thoroughly investigated. Our studies suggest that Smad1 activation may involve cooperation between two TGF-β type I receptors, ALK5 and ALK1 [3], and a co-receptor endoglin [44], thus resembling signaling complex described in endothelial cells [45]. In this study we show for the first time that CCN2 is also required for the activation of the Smad1 pathway. Furthermore, our data suggest that Src/Erk1/2 signaling plays an essential role in this process. This finding is consistent with the previous studies that demonstrated dependence of Smad1 activation on Src in the Angiotensin II-dependent model of diabetic nephropathy [46] and in lung carcinoma [47]. However, the specific role of Src in activation of Smad1 has not been defined.

This study provides new insights into the specific role of CCN2 in the profibrotic TGF-β signaling. We show that CCN2 is required for the TGF-β induced collagen production by facilitating activation of Smad1 and Erk1/2 signaling pathways. Importantly, Smad1 and Erk1/2, as well as Ets1 were shown previously to play a key role in activation of CCN2 gene expression [3], [43], [48], [49]. Together these results indicate the existence of an autocrine loop between TGF-β/Smad1 and CCN2 pathways that regulates pro-fibrotic gene expression in fibroblasts (Fig. 7). Shi-wen et al proposed that an autocrine CCN2-dependent loop maintains fibrosis in scleroderma [50] and several later studies further supported this concept. For example, a strong correlation between collagen and CCN2 expression levels, and Smad1 activation status was observed in hTERT immortalized scleroderma clones [51] and blockade of Smad1 in scleroderma fibroblasts normalized collagen and CCN2 production by these cells [43]. This study provides further details on the signaling cascade leading to activation of Smad1 in SSc fibroblasts and suggests the involvement of Src in this process. Consistent with our findings blockade of Src inhibited collagen production in scleroderma fibroblasts and ameliorated dermal fibrosis in a bleomycin mouse model [34]. Because of its association with tissue fibrosis, CCN2 has been considered an attractive target for anti-fibrotic therapy in SSc and other fibrotic diseases [52]. However, depending on the presence of other factors, CCN2 in addition to having a pro-fibrotic effect, could also play an important role in matrix remodeling. Thus, blockade of its function may only be beneficial during selected stages of the disease. Better understanding of the regulatory mechanisms involved in CCN2 gene expression and function should help to clarify these issues in the future.

## Materials and Methods

### Cell culture

Human fibroblasts cultures were established from the foreskins of the newborn as described previously [49]. Regular maintenance and propagation of the culture was carried out in DMEM supplemented with 10% serum. All experiments were carried out in early passage cells.

### Plasmids, Transient transfections and Luciferase assay

Human collagen promoter (−2 Kb) cloned upstream of luciferase reporter gene in pGL3 vector (COL1A2 -Luc) was a gift from A. Gabrielli, University of Ancona and was described previously [53]. Transient transfections were performed in foreskin fibroblasts seeded into 12 well plates using Fugene6 (Roche) according to the manufacturer's instructions. After overnight incubation some cells were treated with either TGF-β (2.0 ng/ml) or rCCN2 (5–50 ng/ml) (EMP Genetech) and then further incubated for 24 hours. The cells were harvested and assayed for luciferase reporter activity using Promega Luciferase assay kit as per manufacturer's instructions. Co-transfection with pSV-β-galactosidase control vector was used to normalize for transfection efficiency. β-galactosidase was measured using Galacto-Light-Plus (Tropix).

### Adenoviral siRNA vectors

Adenoviral siRNA sequence CCAAGCCTATCAAGTTTGA targeting CCN2 was generated as described previously [49]. Annealed double stranded oligonucleotides were cloned into Mlu1 and Xho 1 sites of pRNAT-H1.1 shuttle vector (Genescript). The shuttle vector with target siRNA sequence was linearized with Pme1 and then electroporated into BJ5183-AD-1 (Stratagene) to generate recombinant Adenoeasy vector. The recombinant Adenoeasy plasmid after linearization with Pac1 enzyme was transfected into QBI-293 cells using Transfectin (Bio-Rad) for generation of adenovirus. The shuttle vector plasmid and Adenoeasy vector plasmid were sequenced to confirm the cloning. The primary adenoviral stock was then amplified and concentrated by Cesium chloride density gradient centrifugation. Control Scrambled adenovirus vector was prepared in the same manner.

SiRNA oligos against Src [47] and β_3_ [54] were ordered from Dharmacon. The oligos were transfected using HiPerfect reagent (Qiagen) as per manufacturer's instructions.

### RNA isolation, QRT-PCR

Total RNA was isolated from the fibroblasts using Tri reagent (MRC Inc) according to manufacturer's instructions. 2 µg of RNA was reverse transcribed in 20 µl reaction volume using random primers and Transcriptor First Strand synthesis kit (Roche) and then diluted to 100 µl. Real time quantitative PCR was carried out using IQ Sybr green mix (Biorad) on Icycler machine (Biorad) using 1 µl of the cDNA in triplicates with β-actin as the internal control. The primers used are CCN2 - 5′-TTGCGAAGCTGACCTGGAAGAGAA 3′ (forward), 5′- AGCTCGGTATGTCTTCATGCTGGT-3′ (reverse); COL1A1 5′- CCAGAAGAACTGGTACATCAGCA -3′ (forward), 5′- CGCCATACTCGAACTGGGAAT -3′ (reverse); COL1A2 5′-GATGTTGAACTTGTTGCTGAGG -3′ (forward), 5′- TCTTTCCCCATTCATTTGTCTT-3′ (reverse) and have been validated to β-actin. The fold change in the levels of genes of interest was determined by 2^−ΔΔCt^. PCR conditions were 95°C for 3 min, followed by 40 cycles of 95°C for 30 sec, 58°C for 1 min. To check for absence of secondary products a melt curve analysis for the PCR product was carried out.

### Western blotting and Immunoprecipitation

Cell lysates were prepared in RIPA buffer. 30–100 µg of protein was separated on SDS-PAGE and then transferred on nitrocellulose membrane. The membranes were blocked with 3% nonfat dry milk in Tween/Tris buffered saline. The membranes were then probed with antibodies against pSmad3, total Smad2/3, pSmad1, pErk, total Erk (Cell Signaling), Total Smad1 (Santa Cruz), and Collagen (Southern Biotechnologies). For CCN2 western blot, the membranes were blocked in 2% gelatin and probed with anti CCN2 antibody (Santa Cruz) overnight at room temperature. Western blotting procedure for phospho-Fli1 and Fli1 was described previously [35]. The blots were then incubated with appropriate horseradish peroxidase coupled secondary antibodies and developed using Chemilumicent Kit (Pierce). For loading control, either the blots were stripped and re-probed for β-actin (Sigma) or separate gels were run and probed.

For immunoprecipitation experiments, cell lysates were prepared in NP40 buffer (50 mM Tris-HCl (pH 8.0), 150 mM NaCl, 0.02% sodium azide, 0.1% SDS, 1% Nonidet P-40, 0.5% sodium deoxycholate, 50 mM sodium fluoride, 0.5 mM dithiothreitol, 2 mM sodium orthovanadate, and 1 mM PMSF with protease inhibitors (Mixture Set III, Calbiochem)) and were precleared with Protein A/G ultralink resin (Pierce). The lysates were then incubated with anti β_3_ antibody (Santa Cruz) or anti α_v_β_3_ antibody (Millipore) overnight at 4°C. The immunocomplexes were then pulled down with Protein A/G ultralink resin and washed 4× with NP40 buffer. The beads were suspended in 2× sample loading buffer and the immunoprecipitates were eluted by boiling for 5 min in 2× SDS sample buffer, and analyzed by Western blot as described earlier.

### Immunohistochemistry

Cells were grown on coverslips and fixed in 4% paraformaldehyde in PBS for 10 min. After washing with PBS, cells were permeabilized with 0.1% Triton X-100 in PBS for 10 min at room temperature. The samples were incubated with anti-α_v_β_3_ (LM609 – Millipore) followed by anti CCN2 antibodies (Biovision) in 1% bovine serum albumin in PBS. Anti-mouse and anti-rabbit conjugated to AlexaFluor 488 and 546 respective secondary antibodies (Invitrogen) were used to visualize the proteins. The coverslips were then mounted on to glass slides and viewed with Olympus IX70 microscope equipped with the Fluoview 300 confocal.

### Statistical analysis

The student's t-test analysis using GraphPad InStat Statistics Software (v 1.12) was performed to determine statistical significance. Values of less than or equal to 0.05 were considered statistically significant.

## Supporting Information

Figure S1 Graphical presentation of densitometric scans. (A) Phosphorylation of Erk in response to TGF-β stimulation after suppression of CCN2 as shown Figure 2B. The values represent mean ± S.E. (n = 3, * p<0.05) (B) phosphorylation of Smad3 in response to TGF-β stimulation after suppression of CCN2 as shown Figure 2C. The values represent mean ± S.E. (n = 3).
(TIF)

Figure S2 α_v_β_3_ integrin mediates TGF-β induced Smad1 phosphorylation (A) Foreskin fibroblasts were transfected with β_3_ siRNA oligos and mRNA levels of β_3_ integrin was measured (n = 3, * p<0.01). (B) Foreskin fibroblasts were pretreated with α_v_β_3_ function blocking antibody LM609 or control IgG and then stimulated with TGF-β for 30 minutes and analyzed for Smad1 phosphorylation.
(TIF)

Figure S3 TGF-β induced Smad1 phosphorylation is mediated through Src (A) Foreskin fibroblasts were transfected with Src siRNA oligos and mRNA levels of Src was measured (n = 3, * p<0.01). (B) Foreskin fibroblasts were pretreated with Src inhibitor SU6656 for 1 hour and then stimulated with TGF-β for 30 minutes and analyzed for Smad1 phosphorylation.
(TIF)

Figure S4 Graphical presentation of densitometric scans of Smad1 protein levels after CCN2 stimulation as shown in Figure 5D. The values represent mean ± S.E. (n = 3, * p<0.05)
(TIF)
