# Supplementary figures and images for: CCN2 Is Required for the TGF-β Induced Activation of Smad1 - Erk1/2 Signaling Network
Source: PLoS One. 2011 Jul 8;6(7):e21911. doi: 10.1371/journal.pone.0021911 (PMC3132735; doi:10.1371/journal.pone.0021911)

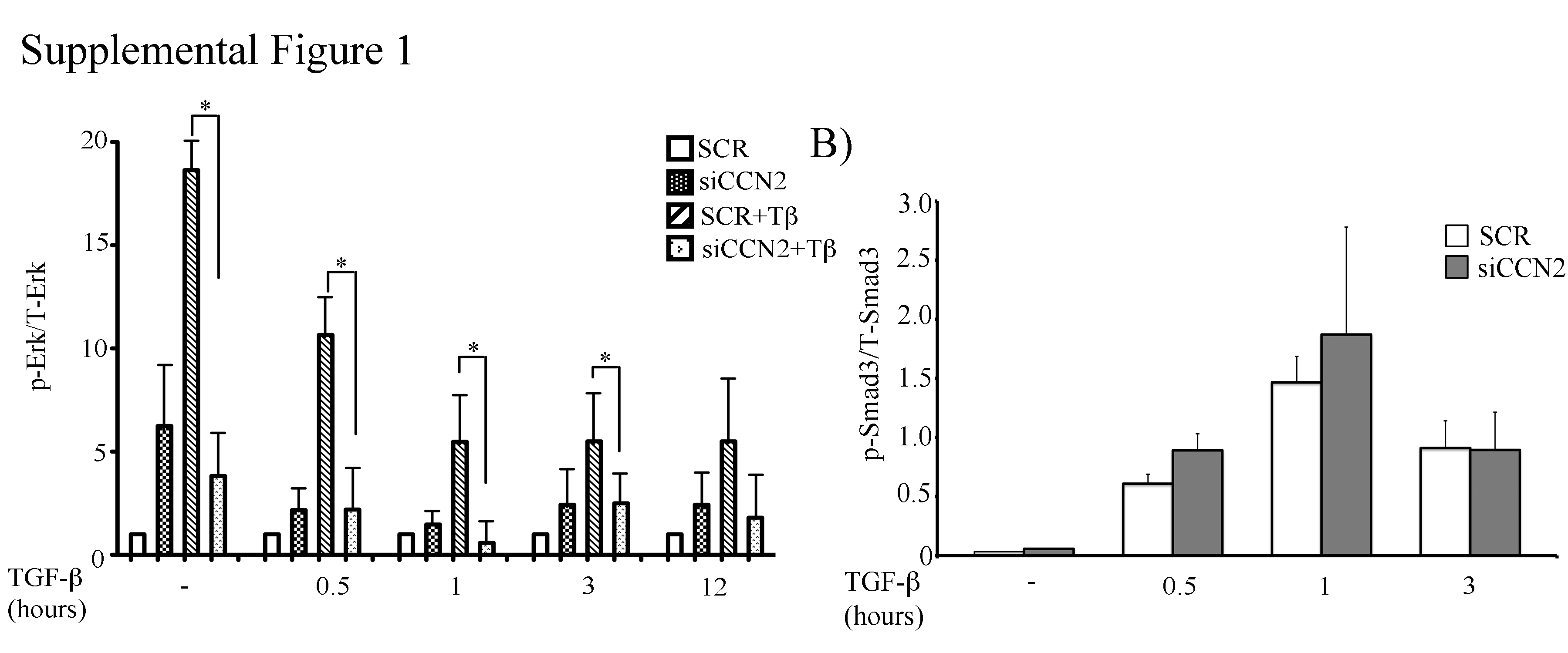

Supplement: Figure S1 — Graphical presentation of densitometric scans. (A) Phosphorylation of Erk in response to TGF-β stimulation after suppression of CCN2 as shown Figure 2B. The values represent mean ± S.E. (n = 3, * p<0.05) (B) phosphorylation of Smad3 in response to TGF-β stimulation after suppression of CCN2 as shown Figure 2C. The values represent mean ± S.E. (n = 3). (TIF) [file pone.0021911.s001.tif]

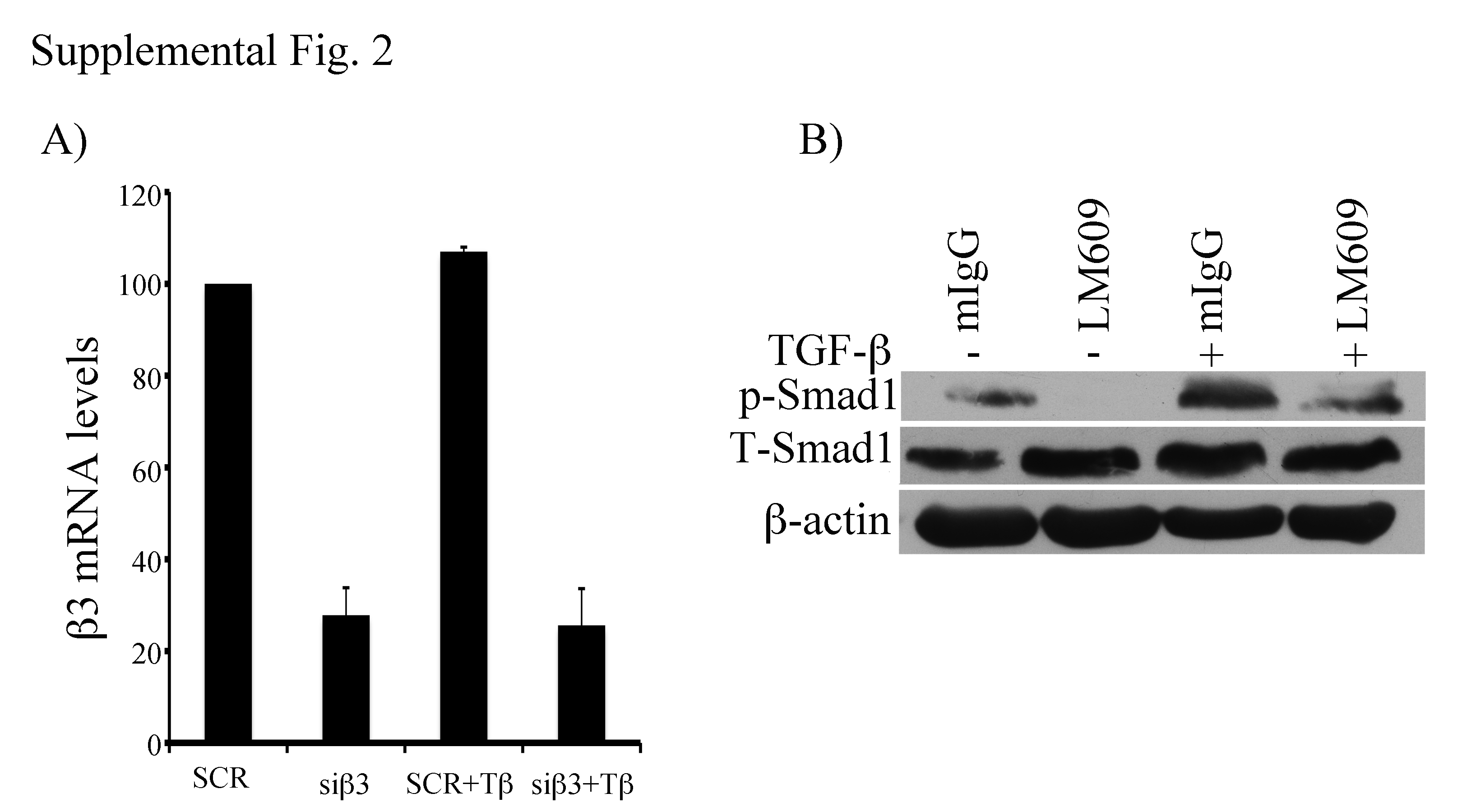

Supplement: Figure S2 — αvβ3 integrin mediates TGF-β induced Smad1 phosphorylation (A) Foreskin fibroblasts were transfected with β3 siRNA oligos and mRNA levels of β3 integrin was measured (n = 3, * p<0.01). (B) Foreskin fibroblasts were pretreated with αvβ3 function blocking antibody LM609 or control IgG and then stimulated with TGF-β for 30 minutes and analyzed for Smad1 phosphorylation. (TIF) [file pone.0021911.s002.tif]

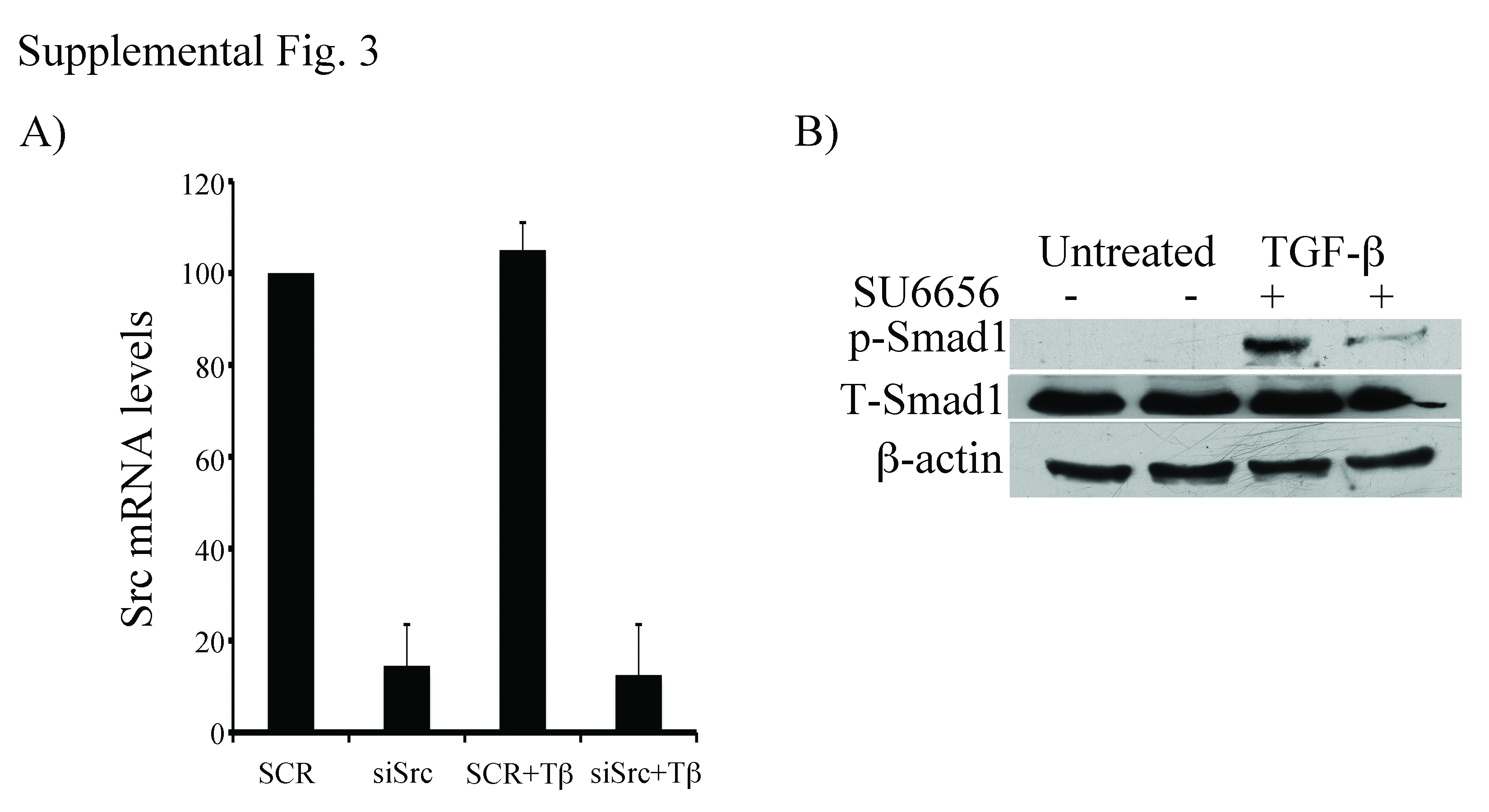

Supplement: Figure S3 — TGF-β induced Smad1 phosphorylation is mediated through Src (A) Foreskin fibroblasts were transfected with Src siRNA oligos and mRNA levels of Src was measured (n = 3, * p<0.01). (B) Foreskin fibroblasts were pretreated with Src inhibitor SU6656 for 1 hour and then stimulated with TGF-β for 30 minutes and analyzed for Smad1 phosphorylation. (TIF) [file pone.0021911.s003.tif]

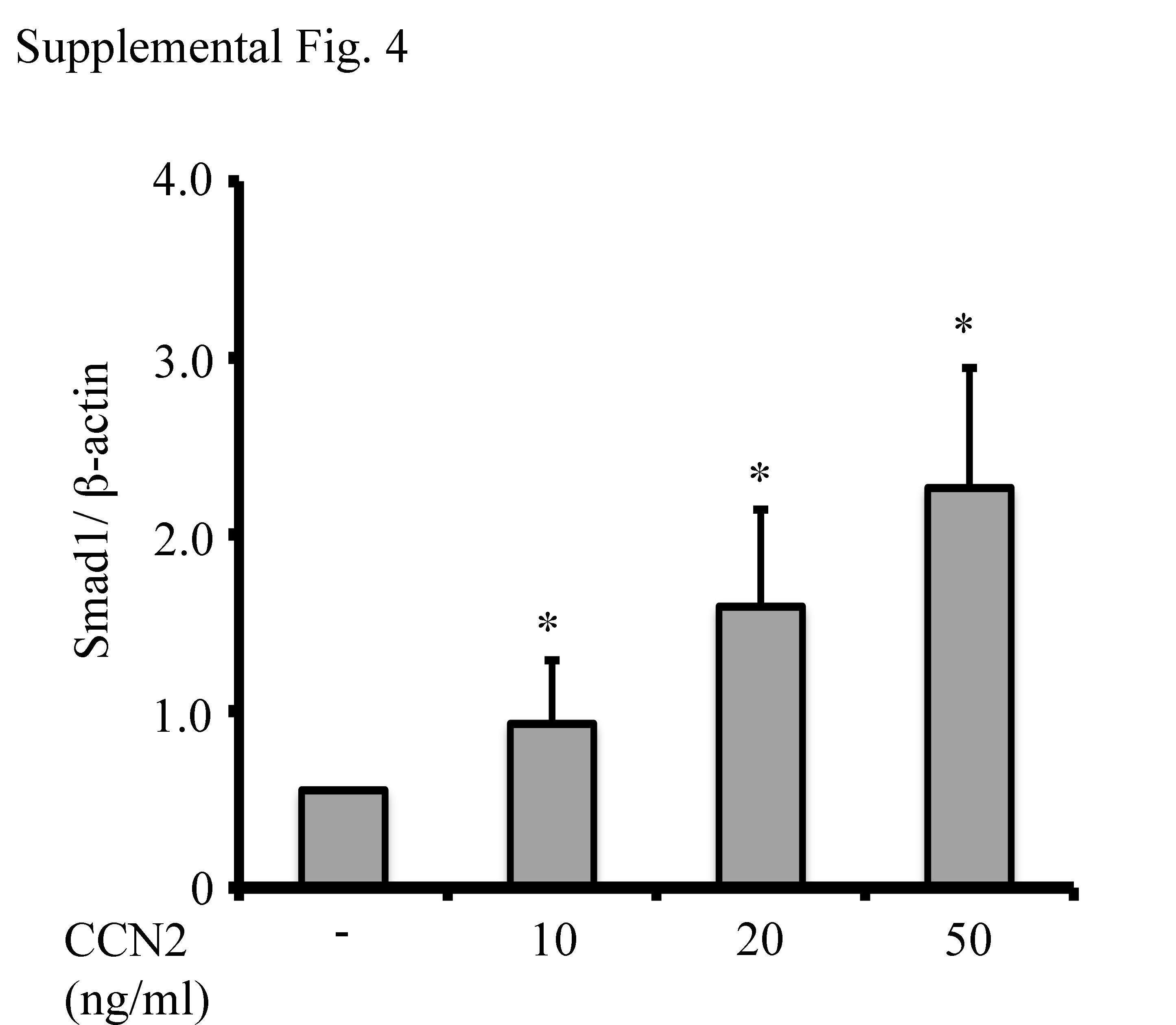

Supplement: Figure S4 — Graphical presentation of densitometric scans of Smad1 protein levels after CCN2 stimulation as shown in Figure 5D. The values represent mean ± S.E. (n = 3, * p<0.05) (TIF) [file pone.0021911.s004.tif]
